# A Comprehensive Review of the Underlying Mechanisms of Resistance and Therapeutic Options for DTR-PA in Bloodstream Infection and Pneumonia: A Clinical Vignette-Based Approach

**DOI:** 10.3390/microorganisms14102203

**Published:** 2026-10-01

**Authors:** Alberto Enrico Maraolo, Roberta Astorri, Paolo Cirillo, Guido Granata, Arianna Emiliozzi, Marco Tescione, Gioconda Brigante, Stefania Cicalini, Nicola Petrosillo, Ivan Gentile, Davide Carcione, Luigi Principe

**Affiliations:** 1 Section of Infectious Diseases, Department of Clinical Medicine and Surgery, University of Naples “Federico II”, 80131 Naples, Italy; ivan.gentile@unina.it; 2 Department of Medicine and Surgery, LUM “Giuseppe Degennaro” University, Casamassima, 70010 Bari, Italy; astorri@lum.it; 3 Systemic and Immune Depression-Associated Infections Unit, National Institute for Infectious Diseases “Lazzaro Spallanzani”, IRCCS, 00149 Rome, Italy; paolo.cirillo@inmi.it (P.C.); arianna.emiliozzi@inmi.it (A.E.); stefania.cicalini@inmi.it (S.C.); 4 Infection Prevention & Control/Infectious Disease Service, Fondazione Policlinico Universitario Campus Bio-Medico, 00127 Rome, Italy; g.granata@policlinicocampus.it (G.G.); n.petrosillo@policlinicocampus.it (N.P.); 5 Department of Anesthesiology and Intensive Care, Grande Ospedale Metropolitano “BMM”, 89124 Reggio di Calabria, Italy; marco.tescione@ospedalerc.it; 6 Laboratory of Clinical Microbiology and Virology, ASST Valle Olona, 21013 Gallarate, Italy; gioconda.brigante@asst-valleolona.it (G.B.); davide.carcione@asst-valleolona.it (D.C.); 7 Clinical Microbiology and Virology Unit, Great Metropolitan Hospital “Bianchi-Melacrino-Morelli”, 89124 Reggio Calabria, Italy; luigi.principe@gmail.com

**Keywords:** *Pseudomonas aeruginosa*, difficult-to-treat resistance, bloodstream infection, pneumonia, cefiderocol, carbapenemase, β-lactam/β-lactamase inhibitor combinations

## Abstract

*Pseudomonas aeruginosa* is a ubiquitous opportunistic pathogen and a leading cause of severe healthcare-associated infections, particularly ventilator-associated pneumonia (VAP) and bloodstream infections (BSIs) in critically ill patients. Its remarkable capacity for both intrinsic and acquired resistance leads to complex phenotypic profiles, culminating in difficult-to-treat resistant *P. aeruginosa* (DTR-PA). Managing DTR-PA presents a daunting clinical challenge characterised by a narrow therapeutic armamentarium, treatment delays, and high attributable mortality. To bridge the gap between complex molecular microbiology and bedside decision-making, this narrative review employs a pragmatic, clinical vignette-based approach. Through six representative fictional scenarios of pneumonia and BSI, we systematically dissect the underlying mechanisms of resistance and their direct therapeutic implications. The vignettes explore combinations of intrinsic adaptations—such as Pseudomonas-derived cephalosporinase (PDC/AmpC) hyperexpression, OprD porin loss, and efflux pump upregulation—as well as the acquisition of serine- (e.g., GES) and metallo-β-lactamases (e.g., VIM, NDM, IMP). Grounded in the updated 2026 Infectious Diseases Society of America (IDSA) guidelines, we evaluate the optimal deployment of newer β-lactam/β-lactamase inhibitor combinations. We highlight the specific preference for ceftolozane–tazobactam in pneumonia, the roles of ceftazidime–avibactam and imipenem–relebactam, and the critical use of cefiderocol for metallo-β-lactamase producers and highly resistant phenotypes. Furthermore, the review addresses pressing clinical controversies: the superiority of targeted monotherapy over historical, toxic combination regimens; the risks of unconditionally applying “shorter-is-better” duration paradigms to DTR-PA; and the alarming frequency of treatment-emergent cross-resistance among novel agents. We also clarify common clinical misconceptions, such as the limited utility of meropenem–vaborbactam and aztreonam–avibactam against specific pseudomonal mechanisms. Ultimately, effective DTR-PA management precludes class-based empirical assumptions. By providing a phenotype-driven bedside aide-mémoire, this review reinforces that rapid recognition, direct agent-specific antimicrobial susceptibility testing (AST), pharmacokinetic/pharmacodynamic (PK/PD)-optimised dosing, and prompt source control remain the absolute cornerstones of survival for patients afflicted by these formidable infections.

## 1. Introduction

*P. aeruginosa* (PA henceforth in combined acronyms) is an aerobic, non-fermenting Gram-negative bacillus with a ubiquitous distribution and a particular affinity for water-rich environments. Its clinical relevance stems from its major role in healthcare-associated infections (HAIs), particularly in critically ill patients, and from its challenging intrinsic and acquired resistance mechanisms, which further complicate treatment in vulnerable hosts [1]. Importantly, *P. aeruginosa* shows a remarkable environmental resistance and a long persistence on surfaces, posing a significant healthcare challenge when medical devices or equipment become contaminated.

*P. aeruginosa* is among the most frequently isolated Gram-negative pathogens and a leading cause of hospital-acquired pneumonia (HAP). Notably, in the European Centre for Disease Prevention and Control (ECDC) Annual Epidemiological Report for HAIs acquired in intensive care units (ICUs), *P. aeruginosa* is confirmed as the most frequently isolated microorganism in ICU-acquired pneumonia in Europe [2], underscoring its prominent role in respiratory infections. It is also frequently involved in central line-associated bloodstream infections (CLABSIs) and catheter-associated urinary tract infections (CAUTIs) [2,3].

While undoubtedly of major importance in HAIs, *P. aeruginosa* is also implicated in community-acquired infections, most often involving the skin and skin structures following water exposure, but also accounting for up to 5% of severe community-acquired pneumonia (CAP) and 1% of community-acquired bloodstream infections (BSIs) [1].

Resistance mechanisms in *P. aeruginosa* involve more than 40 chromosomal genes (approximately 8.4% of which are regulatory), supporting remarkable adaptability to environmental and antimicrobial selective pressure, often through combined mechanisms [4,5]. Intrinsic resistance includes efflux pump expression, production of inactivating enzymes, and downregulation of outer-membrane porins such as OprD. Inducible expression of the intrinsic chromosomal AmpC enzyme is a major determinant of natural resistance to many penicillins and cephalosporins, whereas MexAB-OprM and MexXY contribute to reduced susceptibility to several β-lactams and aminoglycosides. Acquired resistance may arise through mutation or horizontal transfer of mobile genetic elements, while adaptive resistance, including biofilm formation, can be reversible after removal of the environmental stimulus [1,6,7].

For clarity, the intrinsic chromosomal AmpC enzyme is referred to throughout as Pseudomonas-derived cephalosporinase (PDC). Acquired carbapenemases include serine enzymes and metallo-β-lactamases (MBLs). The MBL families most relevant to *P. aeruginosa* include Verona integron-encoded metallo-β-lactamase (VIM), New Delhi metallo-β-lactamase (NDM), and imipenemase (IMP); oxacillinase (OXA)-type enzymes belong to Ambler class D [6].

*P. aeruginosa* resistance patterns vary geographically and over time. Multidrug-resistant (MDR), extensively drug-resistant (XDR), and pandrug-resistant (PDR) describe progressively broader resistance across antimicrobial categories in the framework proposed by Magiorakos et al. [8]. Difficult-to-treat resistance (DTR), introduced by Kadri et al. in 2018, is a separate, treatment-oriented construct reflecting lack of first-line agents with favourable efficacy and toxicity profiles, namely a phenotype featuring intermediate or resistant responses to all reported agents in carbapenem, β-lactam (including aztreonam) and fluoroquinolone categories; it is not a merger of the MDR/XDR/PDR definitions or an additional step after PDR [9]. The IDSA definition of DTR-PA requires non-susceptibility to piperacillin–tazobactam, ceftazidime, cefepime, aztreonam, meropenem, imipenem–cilastatin, ciprofloxacin, and levofloxacin [10]. Carbapenem resistance alone does not establish DTR: a carbapenem-resistant (CR) isolate (CR-PA) may retain activity to a traditional non-carbapenem β-lactam or a fluoroquinolone. Conversely, DTR does not imply resistance to every newer β-lactam or to all antimicrobial categories.

The terminology requires particular care when IDSA is read alongside European Committee on Antimicrobial Susceptibility Testing; (EUCAST) interpretative criteria. EUCAST I means susceptible, increased exposure, and belongs with S among susceptible categories; it is not equivalent to the historical intermediate category or to resistance. For the educational antibiograms in this review, DTR status is conservatively demonstrated by R (resistant) results for all eight defining agents, according to the IDSA definition of DTR-PA. A current EUCAST I result for one of those agents would require separate clinical and pharmacological assessment and must not be counted as R to establish DTR-PA [9,10,11].

The present review uses the 2026 IDSA Guidance, which retains the main 2024 framework while preferring ceftolozane–tazobactam (C/T) for DTR-PA pneumonia and expanding carbapenemase-directed advice and evidence on treatment-emergent resistance [10]. The World Health Organization lists CR-PA as a high-priority research pathogen [12].

*P. aeruginosa* behaves as an opportunistic pathogen, generally affecting patients with structural lung disease (especially in the presence of bronchiectasis, as in chronic obstructive pulmonary disease [COPD] and cystic fibrosis [CF]), burns, or immunosuppression (e.g., neutropenia), with a higher risk for those receiving invasive treatments (e.g., mechanical ventilation or renal replacement therapy) or bearing indwelling vascular and urinary catheters, particularly in the context of prolonged hospitalisation with prior broad-spectrum antimicrobial exposure and/or colonisation. Although it is not typically part of the normal human microbiota, *P. aeruginosa* can behave as either an indolent or a rapidly aggressive coloniser in critically ill patients, frequently causing severe pneumonia (particularly ventilator-associated pneumonia [VAP]), bacteraemia, and sepsis in the ICU setting [13,14].

Therapeutic options for DTR-PA, when supported by antimicrobial susceptibility testing (AST), include the β-lactam/β-lactamase inhibitor (BL/BLI) combinations ceftolozane–tazobactam (C/T), ceftazidime–avibactam (CAZ-AVI), and imipenem–relebactam (IMI-REL), together with cefiderocol; aminoglycosides and polymyxins have more restricted roles [10]. Intravenous fosfomycin has been considered as a companion agent in selected salvage regimens, with limited clinical evidence [14]. Meropenem–vaborbactam (MER-VAB) requires a specific caveat: its activity against *P. aeruginosa* is generally similar to meropenem alone. In the primary study by Castanheira et al., rare isolates with combined MexXY and PDC upregulation had reproducibly lower MER-VAB minimum inhibitory concentrations (MICs). Such exceptions support direct AST, rather than an assumption that vaborbactam overcomes meropenem resistance [15].

Emerging compounds include cefepime–taniborbactam, cefepime–zidebactam, and meropenem–nacubactam, whereas aztreonam–avibactam has limited reliability against MBL-producing *P. aeruginosa* [10,14,16]. Pharmacokinetic/pharmacodynamic (PK/PD)-optimised dosing and prolonged or continuous infusion remain central. Adjunctive inhaled antibiotics are not routinely suggested when an active systemic β-lactam is available, although the 2026 IDSA Guidance allows selective adjunctive use when no newer β-lactam is active and response to systemic therapy is suboptimal [10].

The management of suspected or confirmed DTR-PA infections requires optimal treatment timing and duration, together with a prompt, comprehensive clinical evaluation integrating the infection site and severity, patient stratification, and PK/PD considerations, particularly in critically ill patients. Microbiological confirmation and AST remain cornerstones [10,17].

For infections outside the urinary tract caused by DTR-PA, the 2026 IDSA Guidance identifies C/T, CAZ-AVI, and IMI-REL as preferred agents, with C/T preferred specifically for pneumonia; cefiderocol is an alternative when a preferred β-lactam is inactive or cannot be used [10]. Carbapenemase identification further refines selection. Cefiderocol is preferred for NDM-, VIM-, or IMP-producing *P. aeruginosa*, whereas cefiderocol, CAZ-AVI, or IMI-REL may be considered for *Klebsiella pneumoniae* carbapenemase (KPC)-producing isolates according to AST; C/T is not suggested for KPC producers [10]. Colistin and polymyxin B are constrained by toxicity and should not displace a fully active newer β-lactam [10,18].

The 2026 IDSA Guidance does not suggest routine combination therapy once susceptibility to cefiderocol, CAZ-AVI, C/T, or IMI-REL has been confirmed. Empiric tobramycin plus a newer β-lactam may be reasonable while awaiting AST in patients at high risk of DTR-PA, but the second agent should be discontinued once an active β-lactam is identified. If no β-lactam is active, tobramycin, when susceptible, may be combined with the newer β-lactam whose MIC is closest to its susceptibility breakpoint; if tobramycin susceptibility is not demonstrated, polymyxin B plus a newer β-lactam may be considered. Evidence for these salvage combinations is limited. Treatment-emergent resistance supports repeat AST when infection persists or recurs; the available estimates and their limitations are discussed below [10]. Future strategies include new BL/BLI combinations, small molecules and peptides, bacteriophages and phage-derived lysins, and immunotherapy [14,19].

This manuscript therefore provides a clinically focused synthesis of the mechanisms of resistance and therapeutic options for DTR-PA in BSI and pneumonia through six alternating fictional clinical vignettes.

## 2. Methods

This narrative review integrates microbiological, mechanistic, pharmacological, and therapeutic evidence relevant to DTR-PA bloodstream infection and pneumonia. A narrative design was selected because the questions span several types of evidence and are not suited to a single comparative-effectiveness question. The methodological approach follows principles for transparent narrative reviews [20], and the educational organisation draws on vignette methodology [21] and a published case-based review of antibacterial therapy [22]. Comprehensive reviews of resistant *P. aeruginosa* and current guidance provided the starting framework for the thematic synthesis [1,10,14,16].

A targeted bibliographic update was performed from 1 January 2018, the year of the original DTR report, up to 31 July 2026 using PubMed/MEDLINE as the reference database, supplemented by checks of publisher records and the IDSA and EUCAST websites. Earlier landmark definitions and mechanistic or pharmacological studies were retained through citation tracing. The core PubMed query combined “*Pseudomonas aeruginosa*” with “difficult-to-treat”, “carbapenem-resistant”, or “multidrug-resistant”, and with “pneumonia”, “bacteremia”, “bloodstream”, or “treatment”. Targeted searches and reference checks used the names of the newer agents and the terms “PDC”, “AmpC”, “OprD”, “efflux”, “GES”, “metallo-β-lactamase”, “epithelial lining fluid”, and “aztreonam-avibactam”.

Selection was purposive and based on relevance to the six resistance phenotypes and to invasive infection. Guidelines and guidance documents, clinical trials, systematic reviews, and relevant narrative reviews were prioritised for therapeutic context; comparative observational cohorts were included when trial evidence was lacking. Primary microbiological, animal, and PK/PD studies were retained for claims that could not be established from clinical studies alone. Case reports and small series were used selectively to illustrate rare resistance pathways or salvage strategies. Evidence in organisms other than *P. aeruginosa* was not treated as direct evidence for this species, and mixed MDR/CR/DTR populations were distinguished where relevant. This was a targeted narrative update, without an exhaustive systematic-review search, protocol registration, or formal pooled analysis.

All six patients, isolates, and antibiograms are fictional educational constructs. The categorical results and added MICs illustrate plausible phenotypes; they are not measurements from clinical specimens, estimates of prevalence, or validation of a particular genotype. Moreover, scenario headings are mechanistic teaching labels; unless a molecular result is explicitily reported, they denote hypotheses compatible with the illustrative AST patterns rather than confirmed isolate-level mechanisms. Panels have been aligned to include all eight DTR-defining agents and the four newer β-lactams. EUCAST version 16.1 is the interpretive reference [11]. MICs for BL/BLI combinations refer to the antibacterial component at the specified fixed inhibitor concentration. Cefiderocol requires iron-depleted broth for reference MIC testing. No result is inferred from another drug, and apparent genotype–phenotype discordance would require confirmatory laboratory investigation in clinical practice.

## 3. Scenario 1: PDC Hyperexpression with Efflux and Permeability Changes

### 3.1. Fictional Clinical Vignette: A Case of Pneumonia in a Man with Structural Lung Disease

A 68-year-old man with severe COPD, bilateral bronchiectasis, and repeated courses of antipseudomonal β-lactams was admitted to the ICU with acute respiratory failure and required invasive ventilation. On day 8, after initial improvement while receiving piperacillin–tazobactam for suspected aspiration pneumonia, he developed fever, increased purulent tracheal secretions, worsening oxygenation, and a new right-lower-lobe infiltrate. Bronchoalveolar lavage showed abundant neutrophils and high quantitative growth of *P. aeruginosa*; blood cultures remained negative. Because of his prior colonisation and recent β-lactam exposure, DTR-PA was suspected. The isolate’s susceptibility profile was as follows (Table 1).

**Table 1:** Illustrative antibiogram for Scenario 1.

Antibiotic	Illustrative MIC (mg/L)	Result
Piperacillin–tazobactam	64	R
Ceftazidime–avibactam	2	S
Ceftazidime	32	R
Cefepime	32	R
Ceftolozane–tazobactam	1	S
Imipenem	16	R
Meropenem	16	R
Amikacin	64	R
Aztreonam	64	R
Ciprofloxacin	4	R
Levofloxacin	8	R
Cefiderocol	0.5	S
Colistin	1	*
Imipenem–relebactam	1	S

Fictional educational panel; MICs are illustrative, not measured patient data. S, susceptible, standard dosing regimen; I, susceptible, increased exposure; R, resistant. All eight DTR-defining agents are R. Interpret using EUCAST v16.1 [11]. BL/BLI MICs (mg/L) give the antibacterial component with inhibitor fixed at 4 mg/L for CAZ-AVI, C/T, and IMI-REL. Cefiderocol MICs assume iron-depleted broth microdilution. * Colistin MIC 1 mg/L is below the bracketed 4 mg/L criterion; this excludes phenotypically detectable acquired resistance but does not establish clinical susceptibility for monotherapy. Panels are illustrative and do not prescribe selective laboratory reporting.

### 3.2. Microbiological Notes

This profile combines resistance to traditional antipseudomonal β-lactams and both carbapenems with susceptibility to C/T, CAZ-AVI, and cefiderocol. Hyperproduction of the intrinsic PDC, an ampC enzyme (Ambler class C), can explain resistance to penicillins, cephalosporins, and aztreonam, but is insufficient on its own to explain resistance to both carbapenems. MexAB-OprM upregulation can reduce meropenem exposure; imipenem resistance raises particular concern for concomitant OprD loss or downregulation. Associations between efflux pump expression and carbapenem resistance in clinical isolates do not establish that efflux alone causes the complete phenotype [23,24]. Thus, the vignette represents a plausible combination of chromosomal adaptations, rather than a uniquely identifiable genotype. The older inhibitors clavulanate, sulbactam, and tazobactam do not reliably neutralise derepressed PDC.

AmpC expression is linked to the cell-wall turnover process and is controlled by the LysR-type AmpR transcriptional regulator complexed with certain cell-wall (peptidoglycan)-derived fragments, generically known as muropeptides (mostly anhNAM-P3 in *P. aeruginosa*). Upon muropeptide binding, AmpR changes its conformation to modulate RNA polymerase activity and thus ampC transcription [25,26,27]. Cytosolic muropeptides are further processed by NagZ and the amidase AmpD, and the resulting monosaccharides and peptides are reassembled. Under normal conditions, this complex (UDP-NAM-P5 or UDP-NAM-P3) binds to AmpR and represses ampC expression to a basal level. Conversely, when *P. aeruginosa* strains are exposed to β-lactam inducers (e.g., cefoxitin or piperacillin–tazobactam), the inhibition of penicillin-binding proteins (PBPs), in particular PBP4, alters cell-wall turnover with an increased amount of cytosolic muropeptides, which saturate NagZ and AmpD activity, displacing the repressor UDP-NAM-P5 from AmpR [28,29].

A stable mechanism of AmpC hyperproduction (constitutive rather than induced) is commonly caused by inactivating mutations in ampD or dacB (encoding PBP4) in *P. aeruginosa*, resulting respectively in a lack of UDP-NAM-P5 production or in the promotion of autolysis [25,30,31]. Importantly, AmpC hyperproduction alone in *P. aeruginosa* usually does not confer resistance to the newer cephalosporins (e.g., ceftolozane, cefiderocol) or carbapenems, and it can be overcome by newer inhibitors such as avibactam.

### 3.3. Clinical Perspective

This isolate displays an illustrative DTR-PA phenotype compatible with PDC hyperproduction and additional chromosomal adaptations, yet retains four tested newer options: C/T, CAZ-AVI, IMI-REL and cefiderocol. It is therefore the most therapeutically favourable vignette in this review. The central question is not whether an active agent exists, but which active agent should be prioritised for pneumonia and how exposure should be optimised.

The 2026 IDSA Guidance identifies C/T, CAZ-AVI, and IMI-REL as preferred agents for non-urinary DTR-PA infections and specifically prefers C/T for pneumonia; cefiderocol is an alternative [10]. The 2022 ESCMID guideline supports C/T for severe infections caused by resistant *P. aeruginosa* [18].

For this isolate, C/T and CAZ-AVI are directly supported by AST. C/T is mechanistically attractive because ceftolozane is relatively stable to PDC and less affected than older cephalosporins by common permeability and efflux changes.

No head-to-head randomised clinical outcome trial establishes superiority of C/T over CAZ-AVI in DTR-PA pneumonia. Observational comparative evidence nevertheless contributes to the IDSA preference for C/T at this site. In CACTUS, a matched multicentre study of 420 patients with MDR *P. aeruginosa* pneumonia or bacteraemia, clinical success occurred in 61% of C/T recipients versus 52% of CAZ-AVI recipients (adjusted odds ratio [aOR] 2.07, 95% confidence interval [CI] 1.16–3.70). The association was evident in the pneumonia subgroup (aOR 2.34, 95% CI 1.23–4.47), whereas no difference was identified in the smaller bacteraemia subgroup [32]. A confounder-adjusted meta-analysis also reported higher clinical cure with C/T (OR 1.82, 95% CI 1.10–2.99), particularly in pneumonia (OR 2.48, 95% CI 1.47–4.18) [33]. These findings remain susceptible to residual confounding and differences in treatment selection and outcome assessment.

Differences in pulmonary drug exposure are a possible explanation for the observational findings, but a causal link has not been established. In a primary study of ventilated patients with pneumonia, the epithelial lining fluid (ELF) to unbound-plasma area under the concentration–time curve (AUC) ratio was approximately 50% for ceftolozane after renal-adjusted C/T 3 g every 8 h [34]. In a separate phase 1 study in healthy volunteers, ELF to plasma AUC ratios were 31–32% for ceftazidime and 32–35% for avibactam; elimination from ELF and plasma was similar for both components [35]. These studies differ in population, design, and exposure metrics, so their penetration ratios cannot establish comparative clinical efficacy or prove preferential loss of avibactam exposure.

A 2026 randomised PK study in critically ill patients with nosocomial pneumonia receiving continuous infusions reported median intrapulmonary penetration ratios of 0.66 for ceftolozane, 0.41 for ceftazidime, and 0.44 for avibactam. Both combinations achieved the prespecified ELF targets at standard doses, with substantial variability [36]. This provides direct evidence that adequate joint exposure is achievable for CAZ-AVI and supports individualised dosing; it does not establish comparative clinical superiority. Renal clearance, infusion strategy, MIC, and the chosen PK/PD targets should therefore inform interpretation of the observational pneumonia signal.

The clinical comparisons also require restraint. A GRADE-assessed meta-analysis found no statistically significant differences in clinical success, mortality, microbiological failure, or resistance emergence, with low or very low certainty of evidence [37]; the 200-patient cohort of Almangour et al. likewise reported comparable cure and mortality [38]. Neither CACTUS nor these syntheses established a mortality advantage of C/T over CAZ-AVI [32,33,37,38]. Comparative studies generally favour an active newer β-lactam over historical aminoglycoside- or polymyxin-based regimens, although much of this evidence is observational [39,40]. For the susceptible isolate in this vignette, C/T is a reasonable preferred agent for pneumonia; its selection does not require a claim of proven PK or survival superiority.

Durability of response remains a concern. Treatment can select resistance and cross-resistance, including PDC or PBP3 alterations [41,42]. Susceptibility should be documented at baseline and reassessed if the clinical course falters or infection recurs. In adults with normal renal function, C/T 3 g every 8 h with PK/PD-optimised infusion is appropriate for pneumonia; the dose must be adjusted to renal function. For this susceptible isolate, C/T monotherapy is preferred and routine addition of an aminoglycoside or polymyxin is not supported [10].

## 4. Scenario 2: GES (Guiana Extended-Spectrum β-Lactamase) Production

### 4.1. Fictional Clinical Vignette: A Bloodstream Infection in a Haematological Patient

A 55-year-old woman with acute myeloid leukaemia, prolonged neutropenia, and a tunnelled central venous catheter developed abrupt fever, rigours, hypotension, and an elevated lactate concentration during the third week of hospitalisation. She had received meropenem twice during the preceding month for recurrent febrile neutropenia. Both peripheral and catheter-drawn blood cultures became positive for *P. aeruginosa*, with earlier positivity from the catheter; no pulmonary, urinary, or intra-abdominal focus was identified. The catheter was removed and repeat cultures were obtained. In view of the severe sepsis and extensive prior β-lactam exposure, full antimicrobial susceptibility testing and carbapenemase characterisation were urgently requested. AST yielded the following results (Table 2), corroborated by targeted molecular testing detecting *bla*GES-5.

**Table 2:** Illustrative antibiogram for Scenario 2.

Antibiotic	Illustrative MIC (mg/L)	Result
Piperacillin–tazobactam	128	R
Ceftazidime–avibactam	4	S
Ceftazidime	64	R
Cefepime	64	R
Ceftolozane–tazobactam	16	R
Imipenem	32	R
Meropenem	32	R
Amikacin	64	R
Aztreonam	64	R
Ciprofloxacin	8	R
Levofloxacin	8	R
Cefiderocol	1	S
Colistin	1	*
Imipenem–relebactam	16	R

Fictional educational panel; MICs are illustrative, not measured patient data. S, susceptible, standard dosing regimen; I, susceptible, increased exposure; R, resistant. All eight DTR-defining agents are R. Interpret using EUCAST v16.1 [11]. BL/BLI MICs (mg/L) give the antibacterial component with inhibitor fixed at 4 mg/L for CAZ-AVI, C/T, and IMI-REL. Cefiderocol MICs assume iron-depleted broth microdilution. * Colistin MIC 1 mg/L is below the bracketed 4 mg/L criterion; this excludes phenotypically detectable acquired resistance but does not establish clinical susceptibility for monotherapy. Panels are illustrative and do not prescribe selective laboratory reporting.

### 4.2. Microbiological Notes

This phenotype is characterised by extensive resistance to carbapenems and cephalosporins. Resistance to piperacillin–tazobactam, ceftazidime, and cefepime may be driven, fully or partly, by the hydrolytic activity of GES-5, an Ambler class A serine β-lactamase with carbapenemase activity, although the contribution of other potentially co-existing mechanisms cannot be ruled out. A substitution at Gly170 expands the substrate profile of GES-5 to include imipenem and meropenem [43,44].

C/T resistance reflects the limited protection provided by tazobactam against a GES variant with carbapenemase activity, whereas avibactam can inhibit selected class A serine β-lactamases [44,45]. The GES allele and coexisting mechanisms matter, and CAZ-AVI activity must be demonstrated by AST.

Although aztreonam is stable to MBL hydrolysis, this property does not confer protection against a GES-associated serine β-lactamase phenotype or other co-produced enzymes [23]. Escalation from meropenem to MER-VAB should not be assumed to overcome resistance: Castanheira et al. found generally similar activity, with rare reproducible MIC reductions in selected isolates [15].

Finally, relebactam does not consistently neutralise the carbapenemase action [45] whereas cefiderocol retains activity against the GES phenotype through siderophore-mediated exploitation of bacterial iron-transport systems. This mechanism facilitates periplasmic entry and, together with relative stability to many serine β-lactamases, can preserve activity despite the GES enzyme [46].

### 4.3. Clinical Perspective

The active newer β-lactams in this BSI vignette are CAZ-AVI and cefiderocol, whereas C/T and both carbapenems are resistant. Concurrent carbapenem and C/T resistance is an important red flag for a class A carbapenemase such as GES-5 or for other acquired β-lactamases, and it should prompt carbapenemase characterisation and agent-specific AST rather than class-based inference.

GES-producing *P. aeruginosa* is a challenging DTR phenotype. When CAZ-AVI susceptibility is confirmed, avibactam can inhibit GES and class C enzymes and restore ceftazidime activity; preclinical data support activity against selected GES-producing isolates, but cannot be generalised to every allele or clinical setting [45].

The 2026 IDSA Guidance lists CAZ-AVI among the preferred agents for DTR-PA infections outside the urinary tract [10]. In this isolate, the choice is additionally supported by the specific AST pattern: C/T and carbapenems are resistant, while CAZ-AVI remains susceptible. CAZ-AVI should therefore be prioritised with PK/PD-optimised dosing, alongside source control through catheter removal and documentation of blood-culture clearance.

Cefiderocol remains an important alternative rather than an agent reserved exclusively for MBL producers or salvage therapy. For this CAZ-AVI-susceptible GES phenotype, however, a preferred BL/BLI can be used and cefiderocol can be conserved for intolerance, inactivity, or subsequent resistance. The decisive lesson is that neither meropenem susceptibility nor activity of one newer β-lactam can be inferred from another; each agent requires direct testing [10].

## 5. Scenario 3: PDC Variant/AmpC Hyperexpression plus Efflux Pump Overexpression (CAZ-AVI-Resistant)

### 5.1. Fictional Clinical Vignette: A Case of Ventilator-Associated Pneumonia

A 73-year-old man with post-stroke dysphagia and a tracheostomy remained ventilator-dependent after a prolonged ICU stay. Three weeks earlier, he had completed a course of CAZ-AVI for *P. aeruginosa* VAP, with initial clinical resolution. He subsequently developed a second episode of fever, rising oxygen requirements, purulent secretions, and a new left-basilar consolidation. A protected lower-respiratory-tract sample again yielded *P. aeruginosa*, whereas paired blood cultures were negative. Given the recent exposure to CAZ-AVI and concern for treatment-emergent resistance, the new isolate was tested separately rather than assuming the previous susceptibility pattern. The antibiogram was as follows (Table 3).

**Table 3:** Illustrative antibiogram for Scenario 3.

Antibiotic	Illustrative MIC (mg/L)	Result
Piperacillin–tazobactam	64	R
Ceftazidime–avibactam	32	R
Ceftazidime	64	R
Cefepime	32	R
Ceftolozane–tazobactam	2	S
Imipenem	16	R
Meropenem	16	R
Amikacin	64	R
Aztreonam	64	R
Ciprofloxacin	4	R
Levofloxacin	8	R
Cefiderocol	1	S
Colistin	1	*
Imipenem–relebactam	4	R

Fictional educational panel; MICs are illustrative, not measured patient data. S, susceptible, standard dosing regimen; I, susceptible, increased exposure; R, resistant. All eight DTR-defining agents are R. Interpret using EUCAST v16.1 [11]. BL/BLI MICs (mg/L) give the antibacterial component with inhibitor fixed at 4 mg/L for CAZ-AVI, C/T, and IMI-REL. Cefiderocol MICs assume iron-depleted broth microdilution. * Colistin MIC 1 mg/L is below the bracketed 4 mg/L criterion; this excludes phenotypically detectable acquired resistance but does not establish clinical susceptibility for monotherapy. Panels are illustrative and do not prescribe selective laboratory reporting.

### 5.2. Microbiological Notes

This isolate is resistant to traditional antipseudomonal β-lactams, both carbapenems, and CAZ-AVI, while C/T and cefiderocol remain susceptible. The most likely explanation is PDC hyperproduction and/or an extended-spectrum, inhibitor-resistant PDC variant. More than 500 PDC variants have been described, with amino-acid changes concentrated in the Ω-loop, R2 domain, and H-helix; these alterations can broaden hydrolysis to third- and fourth-generation cephalosporins and reduce inhibitor activity [47,48].

PDC hyperproduction, structural variation, and efflux upregulation can reduce cephalosporin activity, but an antibiogram cannot establish their relative contributions [49]. Additional permeability changes may be needed to explain resistance to both carbapenems. The CAZ-AVI-resistant/C/T-susceptible pattern is possible but isolate-specific: some PDC variants also compromise C/T, and neither the presence of a PDC variant nor prior CAZ-AVI exposure predicts retained C/T activity [50,51,52,53,54].

### 5.3. Clinical Perspective

Unlike Scenario 1, this vignette concerns recurrent pneumonia after CAZ-AVI exposure. The new isolate must undergo independent AST because PDC and other adaptive changes can alter susceptibility to several newer agents [53,54,55].

The reported panel directly supports C/T and cefiderocol. IMI-REL was tested, turning out to be resistant. The 2026 IDSA Guidance prefers C/T for DTR-PA pneumonia and regards cefiderocol as an alternative [10]. Accordingly, PK/PD-optimised C/T monotherapy is the most appropriate choice for this recurrent VAP. Routine adjunctive nebulised therapy is not suggested when an active systemic β-lactam has been demonstrated [10].

Treatment-emergent resistance is central to this vignette. Shah et al. observed resistance more frequently after CAZ-AVI than after C/T in their cohort of MDR-PA bacteraemia or pneumonia (40% versus 10%), with ampC and efflux-regulatory changes among the mechanisms identified [41]. These cohort-specific findings should not be interpreted as universal comparative risks. Repeat cultures and AST are indicated if response is delayed, cultures remain positive, or infection recurs [10].

## 6. Scenario 4: PDC Hyperexpression and OprD Loss

### 6.1. Fictional Clinical Vignette: A Bloodstream Infection in a Patient with Solid Organ Transplantation

A 64-year-old kidney transplant recipient with a chronic ureteral stent and an indwelling urinary catheter presented with fever, flank pain, confusion, and septic shock. During a recent admission, he had received imipinem for a *P. aeruginosa* complicated urinary tract infection. Current blood and urine cultures both grew *P. aeruginosa* with a presumed resistant preliminary phenotype; the urinary catheter was replaced and urgent stent exchange was arranged. A rapid assay did not detect an MBL, despite reduced carbapenem susceptibility. The combination of prior carbapenem exposure, bacteraemia, and a likely high-inoculum urinary source prompted testing of newer BL/BLI combinations. The antibiogram was as follows (Table 4).

**Table 4:** Illustrative antibiogram for Scenario 4.

Antibiotic	Illustrative MIC (mg/L)	Result
Piperacillin–tazobactam	64	R
Ceftazidime–avibactam	16	R
Ceftazidime	32	R
Cefepime	32	R
Ceftolozane–tazobactam	2	S
Imipenem	16	R
Meropenem	16	R
Amikacin	64	R
Aztreonam	64	R
Ciprofloxacin	4	R
Levofloxacin	8	R
Cefiderocol	1	S
Colistin	1	*
Imipenem–relebactam	1	S

Fictional educational panel; MICs are illustrative, not measured patient data. S, susceptible, standard dosing regimen; I, susceptible, increased exposure; R, resistant. All eight DTR-defining agents are R. Interpret using EUCAST v16.1 [11]. BL/BLI MICs (mg/L) give the antibacterial component with inhibitor fixed at 4 mg/L for CAZ-AVI, C/T, and IMI-REL. Cefiderocol MICs assume iron-depleted broth microdilution. * Colistin MIC 1 mg/L is below the bracketed 4 mg/L criterion; this excludes phenotypically detectable acquired resistance but does not establish clinical susceptibility for monotherapy. Panels are illustrative and do not prescribe selective laboratory reporting.

### 6.2. Microbiological Notes

The illustrative phenotype is compatible with PDC hyperexpression plus OprD loss, although it does not prove either mechanism. PDC hyperproduction compromises traditional penicillins and cephalosporins. Since CAZ-AVI resistance was observed, additional mechanisms such as MexAB-OprM overexpression [23], a PDC structural variant, an acquired β-lactamase, or target modification would require investigation [53,56,57]. The pattern of C/T susceptibility associated with CAZ-AVI resistance is rare but not exceptional (less than 2% of strains) [23]. A negative rapid MBL assay excludes only the targets covered by that assay and does not rule out all carbapenemases [58].

Loss or marked reduction in OprD limits imipenem uptake and can contribute to meropenem resistance in combination with PDC hyperexpression and other chromosomal adaptations. C/T can retain activity because ceftolozane is relatively stable to PDC and its entry is not dependent on OprD [59]. Relebactam can restore imipenem activity by inhibiting PDC despite reduced permeability; it does not repair OprD. Clinical-isolate and isogenic-mutant data support retained IMI-REL activity in selected OprD-deficient backgrounds, but direct AST remains necessary [56]. The fluoroquinolone and amikacin resistance shown in the panel requires additional class-specific mechanisms and is not explained by PDC or OprD alone.

### 6.3. Clinical Perspective

This is a plausible non-carbapenemase-mediated phenotype. These MICs must be interpreted against each agent’s own breakpoint and dosing requirements; their absolute numerical values do not rank comparative efficacy.

Relebactam is a diazabicyclooctane inhibitor of class A and class C serine β-lactamases. In a PDC-hyperproducing background, inhibition of periplasmic hydrolysis can restore imipenem activity despite an OprD permeability defect. This provides a mechanistic rationale for considering IMI-REL when the combination itself tests susceptible, rather than inferring its activity from the resistant imipenem result [56].

β-Lactam exposure should be optimised for the selected agent, MIC, renal function, and severity of infection. For imipenem, antibacterial activity relates to the time that the unbound concentration exceeds the MIC; the inhibitor must also achieve adequate exposure. An MIC close to a breakpoint warrants particular attention to dosing, renal replacement therapy, and therapeutic drug monitoring where available. Pulmonary penetration data do not determine drug selection for this bloodstream infection [10].

This is a BSI arising from a complicated urinary source. Catheter replacement, ureteral-stent exchange, and documentation of blood-culture clearance are central to management. C/T and IMI-REL are preferred agents for invasive DTR-PA infection when susceptible [10]. Any of these are reasonable here; IMI-REL illustrates how PDC inhibition can restore activity to a carbapenem combination. The resistant parent carbapenems should not be used. Persistent or recurrent bacteraemia warrants repeat AST rather than assuming that the initial susceptibility profile remains valid.

PK/PD optimisation, prompt source control, and documentation of blood-culture clearance are mandatory. The isolate is also susceptible to cefiderocol, which remains an alternative rather than an agent reserved only for MBL-producing strains. A fully active newer β-lactam should be used as monotherapy; older aminoglycoside- or polymyxin-based combinations add toxicity without demonstrated benefit [10].

## 7. Scenario 5: Metallo-β-Lactamase Production with VIM, NDM, or IMP

### 7.1. Fictional Clinical Vignette: Pneumonia in a Man with Extensive Antimicrobial Exposure

A 60-year-old man with history of bronchiectasis was transferred from a long-term acute-care facility after four weeks of mechanical ventilation and multiple courses of broad-spectrum antibiotics. Surveillance cultures had previously documented colonisation with carbapenem-resistant *P. aeruginosa*. On ICU day 3, he developed fever, worsening hypoxaemia, copious purulent secretions, and a new left-lower-lobe consolidation, followed by vasopressor-dependent shock. Bronchoalveolar lavage showed abundant Gram-negative bacilli and high quantitative growth of *P. aeruginosa*; blood cultures remained negative. A rapid carbapenemase test suggested an MBL-producing strain, raising concern that standard newer BL/BLI combinations would be inactive. The full antibiogram was as follows (Table 5).

**Table 5:** Illustrative antibiogram for Scenario 5.

Antibiotic	Illustrative MIC (mg/L)	Result
Piperacillin–tazobactam	128	R
Ceftazidime–avibactam	64	R
Ceftazidime	64	R
Cefepime	64	R
Ceftolozane–tazobactam	32	R
Imipenem	32	R
Meropenem	32	R
Amikacin	64	R
Aztreonam	128	R
Ciprofloxacin	8	R
Levofloxacin	8	R
Cefiderocol	1	S
Colistin	1	*
Imipenem–relebactam	32	R

Fictional educational panel; MICs are illustrative, not measured patient data. S, susceptible, standard dosing regimen; I, susceptible, increased exposure; R, resistant. All eight DTR-defining agents are R. Interpret using EUCAST v16.1 [11]. BL/BLI MICs (mg/L) give the antibacterial component with inhibitor fixed at 4 mg/L for CAZ-AVI, C/T, and IMI-REL. Cefiderocol MICs assume iron-depleted broth microdilution. * Colistin MIC 1 mg/L is below the bracketed 4 mg/L criterion; this excludes phenotypically detectable acquired resistance but does not establish clinical susceptibility for monotherapy. Panels are illustrative and do not prescribe selective laboratory reporting.

### 7.2. Microbiological Notes

The signal from rapid phenotypic test suggestive of MBL expression together with resistance to CAZ-AVI, C/T, piperacillin–tazobactam, and both carbapenems is highly consistent with an MBL-producing isolate. MBLs use zinc ions for hydrolysis and are not inhibited by avibactam, tazobactam, or relebactam [60,61]. Although aztreonam is intrinsically stable to MBL hydrolysis, co-produced PDC, extended-spectrum β-lactamases (ESBLs), and permeability or efflux barriers commonly render it inactive in *P. aeruginosa* [23].

Cefiderocol can retain activity through siderophore-mediated entry and relative stability to many β-lactamases, including MBLs, but susceptibility must be demonstrated for the individual isolate [62]. OXA-type enzymes are a separate group of Ambler class D serine β-lactamases, not MBLs. Their substrate and inhibitor profiles vary, and not every OXA enzyme is a carbapenemase. Extended-spectrum OXA variants encountered in *P. aeruginosa* can affect newer cephalosporins, including cefiderocol [6,63]. Consequently, an OXA-producing isolate cannot simply be assigned the MBL-directed treatment pathway.

CAZ-AVI inactivity in an MBL-producing isolate reflects the inability of avibactam to inhibit zinc-dependent hydrolysis. Accessory permeability and efflux changes may further reduce exposure [23,60]. For class D OXA enzymes, activity depends on the specific variant and the complete resistance background; genotyping and agent-specific AST are required, rather than extrapolation from OXA-48-producing Enterobacterales or from MBL-producing *P. aeruginosa* [57,63].

In this severely constrained setting, cefiderocol provides a mechanism-directed option by exploiting iron transport for periplasmic entry and retaining activity despite the MBL phenotype. It should be regarded as the preferred active β-lactam for VIM-, NDM-, or IMP-producing *P. aeruginosa* rather than merely as a last-line agent [10,62,64].

### 7.3. Clinical Perspective

MBL-producing *P. aeruginosa* is among the most difficult DTR-PA phenotypes because VIM, NDM, and IMP hydrolyse nearly all conventional β-lactams and are not inhibited by the currently available antipseudomonal BL/BLI partners. Dual CAZ-AVI and C/T resistance is therefore expected, while additional OprD and efflux changes commonly broaden resistance [65].

For confirmed VIM-, NDM-, or IMP-producing *P. aeruginosa*, the 2026 IDSA Guidance identifies cefiderocol as the preferred agent when susceptible [10]. The illustrative cefiderocol MIC of 1 mg/L supports this choice, and monotherapy with renal-adjusted, PK/PD-optimised exposure is appropriate. This mechanism-directed preference applies to MBL producers; treatment of an OXA-producing isolate requires separate interpretation of its enzyme variant and AST.

Colistin remains a salvage option but is limited by nephrotoxicity, neurotoxicity, and less predictable pulmonary exposure. Because cefiderocol is active here, routine combination therapy is not suggested, and C/T, CAZ-AVI, or IMI-REL should not be added in an attempt to treat an MBL phenotype without demonstrated activity [10]. Adjunctive inhaled therapy is likewise not routine when an active systemic β-lactam is available [10].

## 8. Scenario 6: Carbapenemase Production plusCefiderocol Resistance

### 8.1. Fictional Clinical Vignette: A Bloodstream Infection Associated with Septic Shock

A 47-year-old allogeneic haematopoietic stem-cell transplant recipient with profound neutropenia, severe mucositis, and recurrent gastrointestinal colonisation by DTR-PA developed fever and rapidly progressive septic shock. During the previous two months, he had received two courses of cefiderocol for documented *P. aeruginosa* infections. Peripheral and central blood cultures became positive within hours for *P. aeruginosa*; chest imaging showed no pneumonia, and the central line was removed as a possible source. Persistent bacteraemia on repeat cultures raised concern for an extremely resistant subpopulation selected during prior therapy. A rapid molecular assay detected a VIM-type MBL, confirming carbapenemase production. Urgent repeat AST, including cefiderocol and colistin, yielded the following profile (Table 6).

**Table 6:** Illustrative antibiogram for Scenario 6.

Antibiotic	Illustrative MIC (mg/L)	Result
Piperacillin–tazobactam	128	R
Ceftazidime–avibactam	64	R
Ceftazidime	64	R
Cefepime	64	R
Ceftolozane–tazobactam	32	R
Imipenem	32	R
Meropenem	32	R
Amikacin	64	R
Aztreonam	128	R
Ciprofloxacin	8	R
Levofloxacin	8	R
Cefiderocol	8	R
Colistin	1	*
Imipenem–relebactam	32	R

Fictional educational panel; MICs are illustrative, not measured patient data. S, susceptible, standard dosing regimen; I, susceptible, increased exposure; R, resistant. All eight DTR-defining agents are R. Interpret using EUCAST v16.1 [11]. BL/BLI MICs (mg/L) give the antibacterial component with inhibitor fixed at 4 mg/L for CAZ-AVI, C/T, and IMI-REL. Cefiderocol MICs assume iron-depleted broth microdilution. * Colistin MIC 1 mg/L is below the bracketed 4 mg/L criterion; this excludes phenotypically detectable acquired resistance but does not establish clinical susceptibility for monotherapy. Panels are illustrative and do not prescribe selective laboratory reporting.

### 8.2. Microbiological Notes

Cefiderocol has the highest overall likelihood of in vitro activity among currently available newer β-lactams for DTR-PA, making resistance clinically consequential even though it remains uncommon [10,66]. Cefiderocol enters through bacterial iron-uptake systems; resistance can involve altered iron transport, PBP3 changes, and β-lactamase overexpression or structural variation. The relative contribution of each mechanism differs between isolates and cannot be inferred from a resistant MIC alone [46,67,68,69].

Changes in siderophore receptors and associated regulatory pathways, together with PDC or acquired β-lactamase alterations, may compromise cefiderocol activity. Extended-spectrum OXA-2 and OXA-10 derivatives provide a further example in *P. aeruginosa* [63]. Heteroresistance describes a resistant subpopulation within an otherwise susceptible isolate and may complicate routine AST interpretation [70,71]. Emergence of cefiderocol resistance during prolonged treatment of an initially heteroresistant *P. aeruginosa* isolate has been documented [72]. Persistent infection therefore warrants repeat sampling, reliable MIC testing, and consideration of within-isolate diversity.

### 8.3. Clinical Perspective

The displayed isolate is resistant to all tested β-lactams, including C/T, CAZ-AVI, and cefiderocol. This is DTR-PA with very limited documented options, rather than a demonstrated PDR phenotype. The low illustrative colistin MIC indicates absence of phenotypically detectable acquired resistance under the EUCAST bracketed criterion; it is not evidence that colistin monotherapy will be effective.

The detected VIM-type MBL explains the broad resistance to carbapenems and conventional BL/BLI combinations, while additional cefiderocol-resistance mechanisms—such as alteration of iron-transport pathways, PBP3, or PDC—are required to account for cefiderocol non-susceptibility. Aztreonam activity is commonly lost because MBL producers co-express PDC or other serine β-lactamases and may also exhibit efflux-mediated intracellular underexposure.

The added illustrative MICs quantify resistance to the tested newer β-lactams, but IMI-REL and tobramycin results remain unavailable. A complete panel, including reliable cefiderocol MIC determination and combination testing where appropriate, should be obtained urgently. If no β-lactam is active and tobramycin is susceptible, the IDSA Guidance allows tobramycin plus a newer β-lactam selected with reference to its agent-specific breakpoint. If tobramycin susceptibility is not demonstrated, polymyxin B plus a newer β-lactam may be considered, although outcome evidence is limited [10]. The example of an intermediate IMI-REL MIC in the US Guidance should not be relabelled EUCAST I: the breakpoint systems must be applied separately. For a cefiderocol-resistant MBL producer, CAZ-AVI plus aztreonam is a further conditional option when testing supports activity; the MIC of CAZ-AVI alone cannot establish activity of the combination [10,73].

In vitro synergy between polymyxins and fosfomycin has been reported: polymyxin-mediated outer-membrane permeabilisation may facilitate fosfomycin entry, and one study found a 65% synergy rate for fosfomycin plus colistin against MBL-producing *P. aeruginosa* [74]. This experimental signal does not supersede the 2026 IDSA MIC-guided strategy and should not be presented as evidence for routine fosfomycin–polymyxin therapy.

Because this vignette concerns BSI, nebulised colistin has no therapeutic role. Immediate source control, repeat blood cultures, PK/PD-optimised administration, and close toxicity monitoring are essential. If a polymyxin is required outside the urinary tract, the 2026 IDSA Guidance favours polymyxin B over colistin because of its active formulation and more reliable plasma exposure. The colistin MIC should prompt expert laboratory and clinical interpretation and cannot be directly substituted for a polymyxin B susceptibility result [10].

## 9. Discussion

Before turning to the therapeutic implications of these cases, it is worth recalling what DTR-PA actually costs patients. The clinical impact of MDR-PA has been debated for years, since impaired fitness and reduced virulence were expected to offset the effect of resistance, but the balance has shifted decisively towards harm: scarcity of therapeutic options, reliance on less effective and more toxic agents, and an almost unavoidable delay in appropriate targeted therapy translate into longer stays, higher costs and excess mortality, especially in the ICU [75]. Contemporary Italian data quantify this excess. In the prospective nationwide ALARICO cohort of 1276 monomicrobial Gram-negative bloodstream infections, 30-day mortality was 32.8% for CR-PA versus 13.7% in the carbapenem-susceptible Gram-negative BSI comparator group; carbapenem resistance remained independently associated with death after adjustment (adjusted odds ratio 2.99, 95% CI 1.48–5.95), and the mortality attributable to CR-PA was 19% [76]. A separate cohort illustrates the burden of DTR-PA, although its mortality cannot be directly compared with ALARICO because the populations and definitions differ: in a recent single-centre cohort of 51 patients with DTR-PA infections, 30-day all-cause mortality reached 49%, septic shock was the dominant independent predictor of death (adjusted odds ratio 5.52, 95% CI 1.04–29.27), and targeted therapy with CAZ-AVI or C/T showed a favourable but statistically non-significant association with mortality in the final adjusted model (aOR 0.15, 95% CI 0.02–1.17; *p* = 0.070) [77]. These figures explain why expert panels converge on the same operational message that underpins the vignettes presented here: rapid recognition of the resistance phenotype, immediate availability of the newer β-lactams, and early infectious disease consultation are as important as the choice of molecule itself [78].

The six vignettes illustrate a central reality of contemporary DTR-PA management: the therapeutic armamentarium has expanded, but remains narrow and vulnerable to resistance. An AmpC/PDC-hyperproducing isolate may retain several active newer β-lactams, whereas the accumulation of acquired enzymes, PDC structural variants, permeability defects, and cefiderocol-resistance mechanisms can eliminate options. The endpoint is not automatically a colistin-based regimen, but an urgent need for agent-specific MICs and the MIC-guided combination strategy described in the 2026 IDSA Guidance [10]. Emerging BL/BLI combinations and non-traditional approaches such as bacteriophages and phage-derived lysins remain important research priorities. Aztreonam–avibactam, however, should not be portrayed as a dependable future solution for MBL-producing *P. aeruginosa*, because its activity is substantially less reliable than in MBL-producing Enterobacterales [10,14,16,79].

### 9.1. Monotherapy Versus Combination Therapy: When Is Combination Justified?

The 2026 IDSA position is explicit: once cefiderocol, CAZ-AVI, C/T, or IMI-REL is confirmed susceptible, continued combination therapy is not suggested because clinical benefit has not been demonstrated and aminoglycosides or polymyxins add toxicity [10]. This is consistent with comparative evidence showing better outcomes with newer β-lactams than with historical aminoglycoside- or polymyxin-based regimens [39,40]. Empiric tobramycin plus a newer β-lactam may be reasonable as a time-limited bridge in patients at high risk of DTR-PA, but should be de-escalated after AST. Only when no β-lactam is active does the guidance allow an MIC-directed combination: tobramycin, if susceptible, plus the newer β-lactam closest to its breakpoint; otherwise, polymyxin B plus the closest-MIC newer β-lactam [10].

A separate question is whether any companion agent other than tobramycin or a polymyxin deserves consideration when β-lactam options are exhausted, and intravenous fosfomycin is the candidate most often proposed. The 2022 Italian guidelines endorsed by multiple Infectious Disease and Clinical Microbiology societies took an explicit position on this point: combination therapy should not be the routine choice for invasive DTR-PA infections, but may be considered case by case, preferably with infectious disease consultation, and regimens including fosfomycin as the companion agent could be considered (conditional recommendation, low certainty of evidence) [80]. The clinical evidence underpinning that statement comes almost entirely from two Thai retrospective studies. In a 6-year cohort of 136 patients with XDR-PA pneumonia, in which all isolates were susceptible to colistin and/or fosfomycin, active two-drug therapy—most frequently colistin plus fosfomycin (55%) or high-dose 4 h infusion doripenem plus fosfomycin (30%)—was associated with markedly higher 28-day survival and microbiological cure than active monotherapy (90% versus 51% and 90% versus 54%, respectively; both *p* < 0.001), and active monotherapy was an independent predictor of 28-day mortality (adjusted odds ratio 6.63, 95% CI 1.99–22.05), as was the absence of infectious disease consultation (adjusted odds ratio 3.64, 95% CI 1.02–13.01) [81]. The earlier study of 49 patients with CR-PA pneumonia and doripenem MICs of 4–8 mg/L compared two fosfomycin-containing regimens rather than fosfomycin against no fosfomycin, and found essentially superimposable results for high-dose prolonged-infusion doripenem plus fosfomycin and for intravenous colistin plus fosfomycin (clinical cure 60% versus 58%; microbiological cure 72% versus 63%; all-cause mortality 40% versus 42%) [82].

The arguments in favour of fosfomycin are pharmacological rather than clinical. It is a small, highly diffusible molecule that achieves adequate concentrations in lung, bone, cerebrospinal fluid and biofilm, retains activity in acidic and hypoxic environments, acts on an early and unique step of peptidoglycan synthesis and therefore shows no cross-resistance with β-lactams, and displays synergy with β-lactams, carbapenems, polymyxins, aminoglycosides and fluoroquinolones, including with cefiderocol and CAZ-AVI against carbapenemase-producing strains; AUC/MIC is the PD driver, and a loading dose followed by continuous infusion, with high daily doses and therapeutic drug monitoring in life-threatening infection, is increasingly favoured [83]. The arguments against are equally concrete. EUCAST provides no *P. aeruginosa*-specific clinical breakpoints for intravenous fosfomycin—and discourages routine AST for this species; agar dilution is the reference MIC method, but an epidemiological cut-off must not be substituted for a clinical breakpoint; heteroresistance is extremely common and resistance emerges rapidly under monotherapy through glpT and peptidoglycan-recycling pathways, which is precisely why fosfomycin is never proposed alone; reported resistance rates among MDR-PA are high and highly variable (from about 30% up to 50–90% in some series); and the sodium load and risk of hypokalaemia are non-trivial in critically ill patients [11,83]. Above all, the supporting clinical data are retrospective, single-centre, largely predate the newer β-lactams, and are vulnerable to confounding by indication, so that the survival advantage observed for combination therapy may partly reflect the availability of an active agent rather than the act of combining. Fosfomycin is accordingly not part of the MIC-guided algorithm proposed in the 2026 IDSA Guidance, and intravenous formulations are not available in all countries [10]. A reasonable synthesis is that fosfomycin should not be used to build a combination when an active newer β-lactam is available, but that it remains a defensible companion agent—always in combination, always dose-optimised, and ideally with documented in vitro activity or synergy—when no fully active β-lactam exists and the alternative is a polymyxin- or aminoglycoside-based regimen in a patient at high risk of toxicity.

### 9.2. Why MER-VAB Is Not an Escalation from Meropenem in DTR-PA

MER-VAB should not be assumed to broaden meropenem activity against *P. aeruginosa*. The primary investigation by Castanheira et al. found generally similar activity, with rare reproducible reductions in MER-VAB MICs associated with MexXY and PDC upregulation [15]. These results do not establish a clinical advantage or support empirical escalation from meropenem. A lower MIC for a combination also requires interpretation against its own breakpoint and dosing regimen. Direct AST is therefore needed before MER-VAB is considered for a meropenem-resistant isolate.

### 9.3. Is “Shorter” Better for DTR-PA?

The “shorter-is-better” paradigm that has reshaped the duration of therapy for many Gram-negative infections cannot be transposed uncritically to DTR-PA. The pivotal duration trials that underpin short-course recommendations enrolled few, if any, patients with carbapenem-resistant or DTR-PA, and where the relevant subgroup was examined the signal was not reassuring. In the landmark PneumA trial, 8 days of therapy was as effective as 15 days for VAP overall, but recurrence was higher in the subgroup caused by non-fermenting Gram-negative bacilli (40.6% versus 25.4%)—precisely the group to which DTR-PA belongs [84]. The only randomised trial dedicated to *P. aeruginosa* VAP, iDIAPASON, was stopped early for slow accrual and did not establish non-inferiority of 8 versus 15 days: the composite of mortality and recurrence occurred in 35.2% versus 25.5% of patients, with recurrence during the ICU stay at 17% versus 9.2% [85]. Likewise, BALANCE showed that 7 days is non-inferior to 14 days for BSIs, but it excluded severely immunosuppressed patients as well as subjects with foci requiring prolonged treatment, and outcomes were not reported by resistance phenotype [86]. These trials do not establish the optimal duration specifically for DTR-PA. Resistance alone is not an indication for a longer course; immune status, source control, infection site, and response remain the relevant determinants [10].

At the same time, prolonging therapy unnecessarily amplifies both toxicity (notably with polymyxins and aminoglycosides) and selective pressure; in PneumA, multiresistant pathogens emerged less frequently among recurrences in the 8-day arm (42.1% versus 62.0%), a reminder that the trade-off runs in both directions [84]. Until adequately powered trials specifically address duration in DTR-PA, the pragmatic stance is to individualise duration according to source control, infection site, and clinical response rather than to default to a fixed short course. Exposure and duration should be optimised together. Although BLING III did not demonstrate a reduction in 90-day mortality with continuous compared with intermittent β-lactam infusion in sepsis, the companion systematic review and meta-analysis found prolonged infusion to be associated with lower 90-day mortality [87,88]. Because DTR-PA isolates frequently have MICs close to the susceptibility breakpoint even for the agent ultimately selected, prolonged or continuous infusion of the active β-lactam, ideally supported by therapeutic drug monitoring, should be considered within agent-specific dosing recommendations; this does not justify unnecessary prolongation of treatment.

### 9.4. Cross-Resistance and Its Drivers for the Newer BL/BLI Combinations

Cross-resistance among newer BL/BLI combinations limits the assumption that one can automatically replace another. PDC variants can reduce CAZ-AVI and C/T activity, and CAZ-AVI exposure can select ampC and efflux-regulatory changes [41,53,54,89]; PBP3 modification may also affect several agents [42]. In Question 4.5, the 2026 IDSA Guidance summarises treatment-emergent resistance with newer β-lactams at approximately 20%, emergent non-susceptibility during CAZ-AVI or C/T therapy at approximately 24%, and cross-resistance between those two agents above 50% [10]. These refer to different outcomes and source populations: cross-resistance is not an incidence of new resistance in all treated patients. For IMI-REL, the guidance reports no emergent resistance in two trials including 50 patients with *P. aeruginosa* infections, compared with approximately 24% in two observational studies including 46 patients. Differences in case mix, repeat-culture sampling, follow-up, and resistance definitions preclude a direct comparison of drug-specific risks. These estimates support baseline testing of each agent and repeat AST during persistent or recurrent infection, rather than a numerical ranking of agents [10]. Collateral carbapenem sensitivity may occur with some resistance pathways but is not predictable without AST [89].

These cross-resistance patterns also affect the empirical window before definitive AST. International and US surveillance cohorts show incomplete and geographically variable reciprocal susceptibility among C/T, CAZ-AVI, and IMI-REL, while MBL producers are usually resistant to all three [90,91]. Cefiderocol is not immune: prior resistance to C/T or CAZ-AVI has been associated with reduced cefiderocol susceptibility and heteroresistance [92]. In septic shock, cultures, rapid carbapenemase testing, and source control should proceed without delaying immediate antimicrobial therapy [93]. A prior active isolate and local epidemiology should guide the newer β-lactam backbone; when MBL production is strongly suspected, cefiderocol is the most rational empiric β-lactam. A second independently predicted active agent—often tobramycin—may be added briefly, but should be discontinued once identification, carbapenemase results, and agent-specific MICs permit targeted monotherapy [10,94,95].

### 9.5. The Limited Role of Aztreonam–Avibactam in P. aeruginosa

Aztreonam is stable to zinc-dependent MBL hydrolysis, but in *P. aeruginosa* that theoretical advantage is frequently lost because co-produced PDC, ESBLs, or class A/D carbapenemases hydrolyse the monobactam and MexAB-OprM limits intracellular exposure. The 2026 IDSA Guidance therefore does not suggest aztreonam–avibactam for MBL-producing *P. aeruginosa*. CAZ-AVI plus aztreonam is suggested only when cefiderocol resistance precludes cefiderocol use, and the supporting clinical data are limited [10,73]. Aztreonam-based therapy must consequently be contingent on demonstrated in vitro activity and mechanism-aware interpretation, not assumed from the presence of an MBL.

Mauri et al. synthesised 35 in vitro studies and 18 clinical reports, with in vitro data for 2209 MBL-producing Gram-negative isolates. Where MICs were reported, aztreonam with avibactam or CAZ-AVI reached MICs ≤4 mg/L in 79.6% of Enterobacterales, 85.5% of *S. maltophilia*, and 6.2% of *P. aeruginosa* isolates. The *S. maltophilia* estimate came from one report. *P. aeruginosa* accounted for 724 of 808 isolates with low activity or absent synergy (89.6%); this denominator concerns poorly responsive isolates across species, not all *P. aeruginosa* [79]. These are descriptive aggregates from heterogeneous experiments, not a validated *P. aeruginosa* susceptibility rate or a clinical effect estimate. Studies differed in susceptibility and synergy methods, inhibitor concentrations, organism and enzyme distributions, and whether avibactam or CAZ-AVI was combined with aztreonam. Publication and selection bias and sparse pseudomonal clinical data further limit generalisability. The findings support caution but do not alone determine treatment: the recommendation against routine aztreonam–avibactam follows current guidance, while CAZ-AVI plus aztreonam remains a conditional option for cefiderocol-resistant MBL-producing *P. aeruginosa* when activity is demonstrated [10,73,79].

### 9.6. Limitations

This review has the limitations of a narrative, purposively selected evidence synthesis, including possible selection and publication bias and the absence of a formal risk-of-bias assessment. The fictional antibiograms simplify resistance biology for teaching, as done elsewhere [96]. They neither reproduce the full diversity and co-occurrence of mechanisms in clinical isolates nor estimate their real-world frequencies. Phenotypes do not establish genotypes, and the illustrative MICs cannot validate a treatment algorithm. Much of the comparative treatment evidence comes from observational studies with heterogeneous MDR, CR, and DTR definitions, infection sites, dosing, and outcomes. The vignettes therefore cannot substitute for comparative-effectiveness data or for patient-specific microbiology, source control, and PK/PD assessment. Finally, the US IDSA framework and EUCAST interpretive system differ in breakpoints, terminology, and drug availability; these differences must be considered when applying the discussion in other settings.

### 9.7. Summary: Rapid Therapeutic Selection

Table 7 summarises a pragmatic, phenotype-driven approach to initial targeted therapy for pneumonia or BSI due to DTR-PA. It is a bedside aide-mémoire, not a substitute for current AST, agent-specific MICs, molecular characterisation, source control, and patient-specific PK/PD assessment.

**Table 7:** Summary of initial targeted treatment by illustrative phenotype.

Presumed Phenotype	Key Antibiogram Markers	Initial Targeted Approach
PDC hyperexpression with efflux/permeability changes	Traditional β-lactams and carbapenems R; C/T, CAZ-AVI, IMI-REL, cefiderocol S	C/T for pneumonia; CAZ-AVI also active; no routine combination
GES class A carbapenemase	C/T, IMI-REL and carbapenems R; CAZ-AVI and cefiderocol S	CAZ-AVI when susceptible; cefiderocol alternative
PDC variant/hyperexpression with additional adaptations	CAZ-AVI, IMI-REL and carbapenems R; C/T and cefiderocol S	C/T for pneumonia; repeat AST during persistent or recurrent infection
PDC hyperexpression with OprD loss	Carbapenems R; CAZ-AVI, C/T, and IMI-REL S	CAZ-AVI, C/T, or IMI-REL according to AST and patient factors
MBL producer with VIM, NDM, or IMP	CAZ-AVI, C/T, and carbapenems R; cefiderocol S	Cefiderocol when susceptible; OXA enzymes require a separate variant-specific assessment
Cefiderocol-resistant MBL producer	Tested β-lactams R; colistin MIC below bracketed criterion	Complete AST urgently; consider IDSA salvage options, including CAZ-AVI plus aztreonam only when combination activity supports its use

## Data Availability

No new data were created or analyzed in this study. Data sharing is not applicable to this article.

## Abbreviations

AmpC, class C cephalosporinase; anhNAM, 1,6-anhydro-N-acetylmuramic acid; aOR, adjusted odds ratio; AST, antimicrobial susceptibility testing; AUC, area under the concentration–time curve; BL/BLI, β-lactam/β-lactamase inhibitor; BSI, bloodstream infection; C/T, ceftolozane–tazobactam; CAP, community-acquired pneumonia; CAUTI, catheter-associated urinary tract infection; CAZ-AVI, ceftazidime–avibactam; CF, cystic fibrosis; CI, confidence interval; CLABSI, central line-associated bloodstream infection; COPD, chronic obstructive pulmonary disease; CR, carbapenem-resistant; CR-PA, carbapenem-resistant *Pseudomonas aeruginosa*; DTR, difficult-to-treat resistance; DTR-PA, difficult-to-treat resistant *Pseudomonas aeruginosa*; ECDC, European Centre for Disease Prevention and Control; ELF, epithelial lining fluid; ESBL, extended-spectrum β-lactamase; ESCMID, European Society of Clinical Microbiology and Infectious Diseases; EUCAST, European Committee on Antimicrobial Susceptibility Testing; GES, Guiana extended-spectrum β-lactamase; GRADE, Grading of Recommendations Assessment, Development and Evaluation; HAI, healthcare-associated infection; HAP, hospital-acquired pneumonia; I, susceptible, increased exposure; ICU, intensive care unit; IDSA, Infectious Diseases Society of America; IMI-REL, imipenem–relebactam; IMP, imipenemase; KPC, *Klebsiella pneumoniae* carbapenemase; MBL, metallo-β-lactamase; MDR, multidrug-resistant; MDR-PA, multidrug-resistant *Pseudomonas aeruginosa*; MEDLINE, Medical Literature Analysis and Retrieval System Online; MER-VAB, meropenem–vaborbactam; MIC, minimum inhibitory concentration; NDM, New Delhi metallo-β-lactamase; NT, not tested; OprD, outer-membrane porin D; OR, odds ratio; OXA, oxacillinase; PBP, penicillin-binding protein; PDC, Pseudomonas-derived cephalosporinase; PDR, pandrug-resistant; PK/PD, pharmacokinetic/pharmacodynamic; R, resistant; RNA, ribonucleic acid; S, susceptible, standard dosing regimen; UDP-NAM, uridine diphosphate N-acetylmuramic acid; US, United States; VAP, ventilator-associated pneumonia; VIM, Verona integron-encoded metallo-β-lactamase; XDR, extensively drug-resistant; XDR-PA, extensively drug-resistant *Pseudomonas aeruginosa*. P3 and P5 denote tri- and pentapeptide muropeptide forms, respectively; clinical trial names are retained as published.

## References

[1] Vidal-Cortés P., Campos-Fernández S., Cuenca-Fito E., del Río-Carbajo L., Fernández-Ugidos P., López-Ciudad V.J., Nieto-del Olmo J., Rodríguez-Vázquez A., Tizón-Varela A.I. (2025). Difficult-to-treat *Pseudomonas aeruginosa* infections in critically ill patients: A comprehensive review and treatment proposal. Antibiotics.

[2] European Centre for Disease Prevention and Control (2024). Healthcare-associated infections acquired in intensive care units. Annual Epidemiological Report for 2022.

[3] Reynolds D., Kollef M. (2021). The epidemiology and pathogenesis and treatment of *Pseudomonas aeruginosa* infections: An update. Drugs.

[4] Torrens G., van der Schalk T.E., Cortes-Lara S., Timbermont L., del Barrio-Tofiño E., Xavier B.B., Zamorano L., Lammens C., Ali O., Ruzin A. (2022). Susceptibility profiles and resistance genomics of *Pseudomonas aeruginosa* isolates from European ICUs participating in the ASPIRE-ICU trial. J. Antimicrob. Chemother..

[5] Do Rego H., Timsit J.F. (2023). Management strategies for severe *Pseudomonas aeruginosa* infections. Curr. Opin. Infect. Dis..

[6] Botelho J., Grosso F., Peixe L. (2019). Antibiotic resistance in *Pseudomonas aeruginosa*—Mechanisms, epidemiology and evolution. Drug Resist. Updat..

[7] Pang Z., Raudonis R., Glick B.R., Lin T.J., Cheng Z. (2019). Antibiotic resistance in *Pseudomonas aeruginosa*: Mechanisms and alternative therapeutic strategies. Biotechnol. Adv..

[8] Magiorakos A.P., Srinivasan A., Carey R.B., Carmeli Y., Falagas M.E., Giske C.G., Harbarth S., Hindler J.F., Kahlmeter G., Olsson-Liljequist B. (2012). Multidrug-resistant, extensively drug-resistant and pandrug-resistant bacteria: An international expert proposal for interim standard definitions for acquired resistance. Clin. Microbiol. Infect..

[9] Kadri S.S., Adjemian J., Lai Y.L., Spaulding A.B., Ricotta E., Prevots D.R., Palmore T.N., Rhee C., Klompas M., Dekker J.P. (2018). Difficult-to-treat resistance in Gram-negative bacteraemia at 173 US hospitals: Retrospective cohort analysis of prevalence, predictors, and outcome of resistance to all first-line agents. Clin. Infect. Dis..

[10] Tamma P.D., Bonomo R.A., Heil E.L., Justo J.A., Satlin M.J., Mathers A.J. (2026). Infectious Diseases Society of America 2026 guidance on the treatment of antimicrobial-resistant Gram-negative infections. Clin. Infect. Dis..

[11] European Committee on Antimicrobial Susceptibility Testing Breakpoint Tables for Interpretation of MICs and Zone Diameters. Version 16.1, Valid from 24 June to 31 December 2026. https://www.eucast.org/fileadmin/eucast/pdf/breakpoints/v_16.1_Breakpoint_Tables.pdf.

[12] World Health Organization (2024). WHO Bacterial Priority Pathogens List, 2024: Bacterial Pathogens of Public Health Importance to Guide Research, Development and Strategies to Prevent and Control Antimicrobial Resistance.

[13] Alonso B., Fernández-Barat L., Di Domenico E.G., Marín M., Cercenado E., Merino I., de Pablos M., Muñoz P., Guembe M. (2020). Characterization of the virulence of *Pseudomonas aeruginosa* strains causing ventilator-associated pneumonia. BMC Infect. Dis..

[14] Horcajada J.P., Gales A.C., Isler B., Kaye K.S., Kwa A., Landersdorfer C.B., Montero M.M., Oliver A., Pogue J.M., Shields R.K. (2025). Current and future options for the treatment of serious infections due to carbapenem-resistant *Pseudomonas aeruginosa*. Clin. Microbiol. Rev..

[15] Castanheira M., Doyle T.B., Hubler C.M., DeVries S., Shortridge D. (2023). Vaborbactam increases meropenem susceptibility in *Pseudomonas aeruginosa* clinical isolates displaying MexXY and AmpC upregulation. mSphere.

[16] Horcajada J.P., Gales A.C., Isler B., Kaye K.S., Kwa A., Landersdorfer C.B., Montero M.M., Oliver A., Pogue J.M., Shields R.K. (2025). How do I manage difficult-to-treat *Pseudomonas aeruginosa* infections? Key questions for today’s clinicians. Clin. Microbiol. Infect..

[17] Cillóniz C., Dominedò C., Torres A. (2019). An overview of guidelines for the management of hospital-acquired and ventilator-associated pneumonia caused by multidrug-resistant Gram-negative bacteria. Curr. Opin. Infect. Dis..

[18] Paul M., Carrara E., Retamar P., Tängdén T., Bitterman R., Bonomo R.A., de Waele J., Daikos G.L., Akova M., Harbarth S. (2022). European Society of Clinical Microbiology and Infectious Diseases (ESCMID) guidelines for the treatment of infections caused by multidrug-resistant Gram-negative bacilli (endorsed by the European Society of Intensive Care Medicine). Clin. Microbiol. Infect..

[19] González-Pinto L., Alonso-García I., Blanco-Martín T., Camacho-Zamora P., Fraile-Ribot P.A., Outeda-García M., Lasarte-Monterrubio C., Guijarro-Sánchez P., Maceiras R., Moya B. (2024). Impact of chromosomally encoded resistance mechanisms and transferable β-lactamases on the activity of cefiderocol and innovative β-lactam/β-lactamase inhibitor combinations against *Pseudomonas aeruginosa*. J. Antimicrob. Chemother..

[20] Sukhera J. (2022). Narrative reviews: Flexible, rigorous, and practical. J. Grad. Med. Educ..

[21] Evans S.C., Roberts M.C., Keeley J.W., Blossom J.B., Amaro C.M., Garcia A.M., Odar Stough C., Canter K.S., Robles R., Reed G.M. (2015). Vignette methodologies for studying clinicians’ decision-making: Validity, utility, and application in ICD-11 field studies. Int. J. Clin. Health Psychol..

[22] Shif J., Tasissa H., MacDougall C., Heil E.L., Gallagher J.C. (2025). Navigating the muddled middle: A case-based exploration of oral β-lactams for systemic Gram-negative infections. Open Forum Infect. Dis..

[23] Oliver A., Rojo-Molinero E., Arca-Suarez J., Beşli Y., Bogaerts P., Cantón R., Cimen C., Croughs P.D., Denis O., Giske C.G. (2024). *Pseudomonas aeruginosa* antimicrobial susceptibility profiles, resistance mechanisms and international clonal lineages: Update from ESGARS-ESCMID/ISARPAE Group. Clin. Microbiol. Infect..

[24] Khalilipanah S., Ebrahim-Saraie H.S., Nikokar I., Naziri H., Hasannejad-Bibalan M. (2026). Role of MexAB-OprM and MexXY-OprM efflux pumps in carbapenem-resistant *Pseudomonas aeruginosa* from burn wounds. Mol. Biol. Rep..

[25] Torrens G., Hernández S.B., Ayala J.A., Moya B., Juan C., Cava F., Oliver A. (2019). Regulation of AmpC-driven β-lactam resistance in *Pseudomonas aeruginosa*: Different pathways, different signaling. mSystems.

[26] Jacobs C., Huang L.J., Bartowsky E., Normark S., Park J.T. (1994). Bacterial cell wall recycling provides cytosolic muropeptides as effectors for β-lactamase induction. EMBO J..

[27] Jacobs C., Frère J.M., Normark S. (1997). Cytosolic intermediates for cell wall biosynthesis and degradation control inducible β-lactam resistance in Gram-negative bacteria. Cell.

[28] Vadlamani G., Thomas M.D., Patel T.R., Donald L.J., Reeve T.M., Stetefeld J., Standing K.G., Vocadlo D.J., Mark B.L. (2015). The β-lactamase gene regulator AmpR is a tetramer that recognizes and binds the D-Ala-D-Ala motif of its repressor UDP-N-acetylmuramic acid (MurNAc)-pentapeptide. J. Biol. Chem..

[29] Lee M., Dhar S., De Benedetti S., Hesek D., Boggess B., Blázquez B., Mathee K., Mobashery S. (2016). Muropeptides in *Pseudomonas aeruginosa* and their role as elicitors of β-lactam-antibiotic resistance. Angew. Chem. Int. Ed. Engl..

[30] Langaee T.Y., Gagnon L., Huletsky A. (2000). Inactivation of the ampD gene in *Pseudomonas aeruginosa* leads to moderate basal-level and hyperinducible AmpC β-lactamase expression. Antimicrob. Agents Chemother..

[31] Lee M., Hesek D., Blázquez B., Lastochkin E., Boggess B., Fisher J.F., Mobashery S. (2015). Catalytic spectrum of the penicillin-binding protein 4 of *Pseudomonas aeruginosa*, a nexus for the induction of β-lactam antibiotic resistance. J. Am. Chem. Soc..

[32] Shields R.K., Abbo L.M., Ackley R., Aitken S.L., Albrecht B., Babiker A., Burgoon R.A., Cifuentes R., Claeys K.C., Curry S.R. (2025). Effectiveness of ceftazidime-avibactam versus ceftolozane-tazobactam for multidrug-resistant *Pseudomonas aeruginosa* infections in the USA (CACTUS): A multicentre, retrospective, observational study. Lancet Infect. Dis..

[33] Gatti M., De Paola R., Giorgi B., Pea F. (2026). Comparison of ceftolozane-tazobactam and ceftazidime-avibactam in the treatment of multidrug-resistant/difficult-to-treat resistant *Pseudomonas aeruginosa* infections: A systematic review and meta-analysis. Clin. Infect. Dis..

[34] Caro L., Nicolau D.P., De Waele J.J., Kuti J.L., Larson K.B., Gadzicki E., Yu B., Zeng Z., Adedoyin A., Rhee E.G. (2020). Lung penetration, bronchopulmonary pharmacokinetic/pharmacodynamic profile and safety of 3 g of ceftolozane/tazobactam administered to ventilated, critically ill patients with pneumonia. J. Antimicrob. Chemother..

[35] Nicolau D.P., Siew L., Armstrong J., Li J., Edeki T., Learoyd M., Das S. (2015). Phase 1 study assessing the steady-state concentration of ceftazidime and avibactam in plasma and epithelial lining fluid following two dosing regimens. J. Antimicrob. Chemother..

[36] Benítez-Cano A., Sancho-Araiz A., Carazo J., Ramos I., Sánchez-Font A., Chalela R., López-Palacio M., García-Prieto F.F., Navarrete-Rouco M.E., Juanes-Borrego A.M. (2026). Intrapulmonary penetration of ceftolozane/tazobactam and ceftazidime/avibactam administered by continuous infusion in critically ill patients with nosocomial pneumonia: A randomized pharmacokinetic trial. Crit. Care.

[37] Shah A., Rizvi S.V.Z., Nawaz J., Saif H., Rizvi S.U.A., Rathi D., Fatima S., Khan S., Khalid A.A., Al Badri S.G. (2026). Comparative efficacy of ceftolozane-tazobactam versus ceftazidime-avibactam for *Pseudomonas aeruginosa* infections: A GRADE-assessed systematic review and meta-analysis. Ann. Med. Surg..

[38] Almangour T.A., Ghonem L., Alassiri D., Aljurbua A., Al Musawa M., Alharbi A., Almohaizeie A., Almuhisen S., Alghaith J., Damfu N. (2023). Ceftolozane-tazobactam versus ceftazidime-avibactam for the treatment of infections caused by multidrug-resistant *Pseudomonas aeruginosa*: A multicenter cohort study. Antimicrob. Agents Chemother..

[39] Collings H., Stone M., Chrysostomou M., Hirst A., Waldenberg T.E., Yucel E., Lodise T. (2026). A systematic literature review and (network) meta-analysis of the effectiveness of ceftolozane/tazobactam versus aminoglycosides/polymyxins and ceftazidime/avibactam for treating adult patients with multidrug-resistant *Pseudomonas aeruginosa* infections. Antimicrob. Agents Chemother..

[40] Lodise T.P., Min J., Nathanson B.H., Yücel E. (2025). Comparison of early treatment with ceftolozane/tazobactam versus polymyxin-based therapy of pneumonia due to MDR *Pseudomonas aeruginosa* (PUMA). Antimicrob. Agents Chemother..

[41] Shah S., Kline E.G., Haidar G., Squires K.M., Pogue J.M., McCreary E.K., Ludwig J., Clarke L.G., Stellfox M., Van Tyne D. (2025). Rates of resistance to ceftazidime-avibactam and ceftolozane-tazobactam among patients treated for multidrug-resistant *Pseudomonas aeruginosa* bacteraemia or pneumonia. Clin. Infect. Dis..

[42] Rapsinski G.J., Rokes A.B., Van Tyne D., Cooper V.S. (2025). Mutations leading to ceftolozane/tazobactam and imipenem/cilastatin/relebactam resistance during in vivo exposure to ceftazidime/avibactam in *Pseudomonas aeruginosa*. Microbiol. Spectr..

[43] Poirel L., Le Thomas I., Naas T., Karim A., Nordmann P. (2000). Biochemical sequence analyses of GES-1, a novel class A extended-spectrum β-lactamase, and the class 1 integron In52 from *Klebsiella pneumoniae*. Antimicrob. Agents Chemother..

[44] Stewart N.K., Smith C.A., Frase H., Black D.J., Vakulenko S.B. (2015). Kinetic and structural requirements for carbapenemase activity in GES-type β-lactamases. Biochemistry.

[45] Abouelhassan Y., Gill C.M., Nicolau D.P. (2023). Assessing the in vivo efficacy of rational antibiotics and combinations against difficult-to-treat *Pseudomonas aeruginosa* producing GES β-lactamases. J. Antimicrob. Chemother..

[46] Shields R.K., Kline E.G., Squires K.M., Van Tyne D., Doi Y. (2023). In vitro activity of cefiderocol against *Pseudomonas aeruginosa* demonstrating evolved resistance to novel β-lactam/β-lactamase inhibitors. JAC Antimicrob. Resist..

[47] Karthik M., Kacha S., Rajendran S., Bakthavatchalam Y.D., Lal B., Walia K., Veeraraghavan B. (2024). Genetic characteristics and diversity of PDC variants of *Pseudomonas aeruginosa* and its clinical relevance. Infect. Genet. Evol..

[48] Langendonk R.F., Neill D.R., Fothergill J.L. (2021). The building blocks of antimicrobial resistance in *Pseudomonas aeruginosa*: Implications for current resistance-breaking therapies. Front. Cell. Infect. Microbiol..

[49] Castanheira M., Mills J.C., Farrell D.J., Jones R.N. (2014). Mutation-driven β-lactam resistance mechanisms among contemporary ceftazidime-nonsusceptible *Pseudomonas aeruginosa* isolates from U.S. hospitals. Antimicrob. Agents Chemother..

[50] Sid Ahmed M.A., Khan F.A., Hadi H.A., Skariah S., Sultan A.A., Salam A., Al Khal A.L., Söderquist B., Ibrahim E.B., Omrani A.S. (2022). Association of blaVIM-2, blaPDC-35, blaOXA-10, blaOXA-488 and blaVEB-9 β-lactamase genes with resistance to ceftazidime–avibactam and ceftolozane–tazobactam in multidrug-resistant *Pseudomonas aeruginosa*. Antibiotics.

[51] Arca-Suárez J., Vázquez-Ucha J.C., Fraile-Ribot P.A., Lence E., Cabot G., Martínez-Guitián M., Lasarte-Monterrubio C., Rodríguez-Iglesias M., Beceiro A., González-Bello C. (2020). Molecular and biochemical insights into the in vivo evolution of AmpC-mediated resistance to ceftolozane/tazobactam during treatment of an MDR *Pseudomonas aeruginosa* infection. J. Antimicrob. Chemother..

[52] Berrazeg M., Jeannot K., Ntsogo Enguéné V.Y., Broutin I., Loeffert S., Fournier D., Plésiat P. (2015). Mutations in β-lactamase AmpC increase resistance of *Pseudomonas aeruginosa* isolates to antipseudomonal cephalosporins. Antimicrob. Agents Chemother..

[53] Slater C.L., Winogrodzki J., Fraile-Ribot P.A., Oliver A., Khajehpour M., Mark B.L. (2020). Adding insult to injury: Mechanistic basis for how AmpC mutations allow *Pseudomonas aeruginosa* to accelerate cephalosporin hydrolysis and evade avibactam. Antimicrob. Agents Chemother..

[54] Barnes M.D., Taracila M.A., Rutter J.D., Bethel C.R., Galdadas I., Hujer A.M., Caselli E., Prati F., Dekker J.P., Papp-Wallace K.M. (2018). Deciphering the evolution of cephalosporin resistance to ceftolozane-tazobactam in *Pseudomonas aeruginosa*. mBio.

[55] Dorazio A.J., Kline E.G., Squires K.M., Shah S., Van Tyne D., Wu J.Y., Pogue J.M., Shields R.K. (2026). Ceftazidime-avibactam resistance evolution in *Pseudomonas aeruginosa* and implications for cross-resistance to other novel β-lactams. Antimicrob. Agents Chemother..

[56] Fraile-Ribot P.A., Zamorano L., Orellana R., del Barrio-Tofiño E., Sánchez-Diener I., Cortes-Lara S., López-Causapé C., Cabot G., Bou G., Martínez-Martínez L. (2020). Activity of imipenem-relebactam against a large collection of *Pseudomonas aeruginosa* clinical isolates and isogenic β-lactam-resistant mutants. Antimicrob. Agents Chemother..

[57] Torrens G., Cabot G., Ocampo-Sosa A.A., Conejo M.C., Zamorano L., Navarro F., Pascual A., Martínez-Martínez L., Oliver A. (2016). Activity of ceftazidime-avibactam against clinical and isogenic laboratory *Pseudomonas aeruginosa* isolates expressing combinations of most relevant β-lactam resistance mechanisms. Antimicrob. Agents Chemother..

[58] Tenover F.C., Nicolau D.P., Gill C.M. (2022). Carbapenemase-producing *Pseudomonas aeruginosa*—An emerging challenge. Emerg. Microbes Infect..

[59] Moyá B., Zamorano L., Juan C., Pérez J.L., Ge Y., Oliver A. (2010). Activity of a new cephalosporin, CXA-101 (FR264205), against β-lactam-resistant *Pseudomonas aeruginosa* mutants selected in vitro and after antipseudomonal treatment of intensive care unit patients. Antimicrob. Agents Chemother..

[60] Mojica M.F., Rossi M.A., Vila A.J., Bonomo R.A. (2022). The urgent need for metallo-β-lactamase inhibitors: An unattended global threat. Lancet Infect. Dis..

[61] Hong D.J., Bae I.K., Jang I.H., Jeong S.H., Kang H.K., Lee K. (2015). Epidemiology and characteristics of metallo-β-lactamase-producing *Pseudomonas aeruginosa*. Infect. Chemother..

[62] Takemura M., Wise M.G., Hackel M.A., Sahm D.F., Yamano Y. (2023). In vitro activity of cefiderocol against MBL-producing Gram-negative bacteria collected in North America and Europe in five consecutive annual multinational SIDERO-WT surveillance studies (2014–2019). J. Antimicrob. Chemother..

[63] Vuillemin X., Da Silva M., Bour M., Landon C., Plésiat P., Jeannot K. (2023). Cefiderocol activity is compromised by acquired extended-spectrum oxacillinases in *Pseudomonas aeruginosa*. Int. J. Antimicrob. Agents..

[64] Karlowsky J.A., Hackel M.A., Takemura M., Yamano Y., Echols R., Sahm D.F. (2022). In vitro susceptibility of Gram-negative pathogens to cefiderocol in five consecutive annual multinational SIDERO-WT surveillance studies, 2014 to 2019. Antimicrob. Agents Chemother..

[65] Li J., Wang J., Wu W., Li Z., Wu X., Ren J. (2025). The diversity of carbapenemases in *Pseudomonas aeruginosa*: A global genomic epidemiology investigation. Clin. Microbiol. Infect..

[66] Karakonstantis S., Rousaki M., Vassilopoulou L., Kritsotakis E.I. (2024). Global prevalence of cefiderocol non-susceptibility in Enterobacterales, *Pseudomonas aeruginosa*, *Acinetobacter baumannii*, and *Stenotrophomonas maltophilia*: A systematic review and meta-analysis. Clin. Microbiol. Infect..

[67] Karakonstantis S., Rousaki M., Kritsotakis E.I. (2022). Cefiderocol: Systematic review of mechanisms of resistance, heteroresistance and in vivo emergence of resistance. Antibiotics.

[68] Wang L., Zhu J., Chen L., Du H. (2024). Cefiderocol: Clinical application and emergence of resistance. Drug Resist. Updat..

[69] Simner P.J., Beisken S., Bergman Y., Posch A.E., Cosgrove S.E., Tamma P.D. (2021). Cefiderocol activity against clinical *Pseudomonas aeruginosa* isolates exhibiting ceftolozane-tazobactam resistance. Open Forum Infect. Dis..

[70] Band V.I., Weiss D.S. (2019). Heteroresistance: A cause of unexplained antibiotic treatment failure?. PLoS Pathog..

[71] Nicoloff H., Hjort K., Levin B.R., Andersson D.I. (2019). The high prevalence of antibiotic heteroresistance in pathogenic bacteria is mainly caused by gene amplification. Nat. Microbiol..

[72] Teran N., Egge S.L., Phe K., Baptista R.P., Tam V.H., Miller W.R. (2024). The emergence of cefiderocol resistance in *Pseudomonas aeruginosa* from a heteroresistant isolate during prolonged therapy. Antimicrob. Agents Chemother..

[73] Montero M.M., Domene-Ochoa S., Prim N., Ferrer-González E., Sánchez-Díaz A.M., López-Causapé C., Vega-Toribio V., Sorlí L., Luque S., Padilla E. (2025). Addressing carbapenemase-producing extensively drug-resistant *Pseudomonas aeruginosa*: The potential of cefiderocol and ceftazidime-avibactam plus aztreonam therapy. Eur. J. Clin. Microbiol. Infect. Dis..

[74] Wohlfarth E., Dinh A., Vrioni G., Żabicka D., Bernardo M., Tascini C., Noussair L., Mayer C. (2025). In vitro evaluation of fosfomycin combinations against metallo-β-lactamase-producing *Klebsiella pneumoniae* and *Pseudomonas aeruginosa* clinical isolates. Antibiotics.

[75] Maraolo A.E., Cascella M., Corcione S., Cuomo A., Nappa S., Borgia G., De Rosa F.G., Gentile I. (2017). Management of multidrug-resistant *Pseudomonas aeruginosa* in the intensive care unit: State of the art. Expert. Rev. Anti Infect. Ther..

[76] Falcone M., Tiseo G., Carbonara S., Marino A., Di Caprio G., Carretta A., Mularoni A., Mariani M.F., Maraolo A.E., Scotto R. (2023). Mortality attributable to bloodstream infections caused by different carbapenem-resistant Gram-negative bacilli: Results from a nationwide study in Italy (ALARICO Network). Clin. Infect. Dis..

[77] Maraolo A.E., Gallicchio A., Fotticchia V., Catania M.R., Scotto R., Gentile I. (2026). Clinical outcomes and predictors of mortality in patients with difficult-to-treat resistant *Pseudomonas aeruginosa* infections: A retrospective cohort study. Antibiotics.

[78] Coppola N., Maraolo A.E., Onorato L., Scotto R., Calò F., Atripaldi L., Borrelli A., Corcione A., De Cristofaro M.G., Durante-Mangoni E. (2022). Epidemiology, mechanisms of resistance and treatment algorithm for infections due to carbapenem-resistant Gram-negative bacteria: An expert panel opinion. Antibiotics.

[79] Mauri C., Maraolo A.E., Di Bella S., Luzzaro F., Principe L. (2021). The revival of aztreonam in combination with avibactam against metallo-β-lactamase-producing Gram-negatives: A systematic review of in vitro studies and clinical cases. Antibiotics.

[80] Tiseo G., Brigante G., Giacobbe D.R., Maraolo A.E., Gona F., Falcone M., Giannella M., Grossi P., Pea F., Rossolini G.M. (2022). Diagnosis and management of infections caused by multidrug-resistant bacteria: Guideline endorsed by the Italian Society of Infection and Tropical Diseases (SIMIT), the Italian Society of Anti-Infective Therapy (SITA), the Italian Group for Antimicrobial Stewardship (GISA), the Italian Association of Clinical Microbiologists (AMCLI) and the Italian Society of Microbiology (SIM). Int. J. Antimicrob. Agents.

[81] Khawcharoenporn T., Chuncharunee A., Maluangnon C., Taweesakulvashra T., Tiamsak P. (2018). Active monotherapy and combination therapy for extensively drug-resistant *Pseudomonas aeruginosa* pneumonia. Int. J. Antimicrob. Agents.

[82] Apisarnthanarak A., Mundy L.M. (2012). Carbapenem-resistant *Pseudomonas aeruginosa* pneumonia with intermediate minimum inhibitory concentrations to doripenem: Combination therapy with high-dose, 4-h infusion of doripenem plus fosfomycin versus intravenous colistin plus fosfomycin. Int. J. Antimicrob. Agents.

[83] Pipitone G., Di Bella S., Maraolo A.E., Granata G., Gatti M., Principe L., Russo A., Gizzi A., Pallone R., Cascio A. (2023). Intravenous fosfomycin for systemic multidrug-resistant *Pseudomonas aeruginosa* infections. Antibiotics.

[84] Chastre J., Wolff M., Fagon J.Y., Chevret S., Thomas F., Wermert D., Clementi E., Gonzalez J., Jusserand D., Asfar P. (2003). Comparison of 8 vs. 15 days of antibiotic therapy for ventilator-associated pneumonia in adults: A randomized trial. JAMA.

[85] Bouglé A., Tuffet S., Federici L., Leone M., Monsel A., Dessalle T., Amour J., Dahyot-Fizelier C., Barbier F., Luyt C.E. (2022). Comparison of 8 versus 15 days of antibiotic therapy for *Pseudomonas aeruginosa* ventilator-associated pneumonia in adults: A randomized, controlled, open-label trial. Intensive Care Med..

[86] Daneman N., Rishu A., Pinto R., A Rogers B., Shehabi Y., Parke R., Cook D., Arabi Y., Muscedere J., BALANCE Investigators, for the Canadian Critical Care Trials Group, the Association of Medical Microbiology and In-fectious Disease Canada Clinical Research Network, the Australian and New Zealand Intensive Care Society Clinical Trials Group, and the Austr (2025). Antibiotic treatment for 7 versus 14 days in patients with bloodstream infections. N. Engl. J. Med..

[87] Dulhunty J.M., Brett S.J., De Waele J.J., Rajbhandari D., Billot L., Cotta M.O., Davis J.S., Finfer S., Hammond N.E., Knowles S. (2024). Continuous vs. intermittent β-lactam antibiotic infusions in critically ill patients with sepsis: The BLING III randomized clinical trial. JAMA.

[88] Abdul-Aziz M.H., Hammond N.E., Brett S.J., Cotta M.O., De Waele J.J., Devaux A., Di Tanna G.L., Dulhunty J.M., Elkady H., Eriksson L. (2024). Prolonged vs. intermittent infusions of β-lactam antibiotics in adults with sepsis or septic shock: A systematic review and meta-analysis. JAMA.

[89] Hernando-Amado S., Gomis-Font M.A., Valverde J.R., López-Causapé C., Oliver A., Martínez J.L. (2025). Ceftazidime-avibactam use selects multidrug resistance and prevents designing collateral-sensitivity-based therapies against *Pseudomonas aeruginosa*. Nat. Commun..

[90] Gottesdiener L.S., Li Y., Greenwood-Quaintance K.E., Komarow L., Arias C.A., Cober E., Herc E.S., Kaye K.S., Huskins W.C., Figueroa J. (2026). Resistance to novel β-lactam/β-lactamase inhibitors among carbapenem-resistant *Pseudomonas aeruginosa* and clinical implications in the prospective observational Pseudomonas study. Antimicrob. Agents Chemother..

[91] Karlowsky J.A., Lob S.H., DeRyke C.A., Siddiqui F., Young K., Motyl M.R., Sahm D.F. (2022). In vitro activity of ceftolozane-tazobactam, imipenem-relebactam, ceftazidime-avibactam, and comparators against *Pseudomonas aeruginosa* isolates collected in United States hospitals according to results from the SMART Surveillance Program, 2018 to 2020. Antimicrob. Agents Chemother..

[92] Egge S.L., Kang D., Pouya N., Thornton P.C., Flores E., Clarke K., Adelman M.W., Rosenbaum C.L., Baptista R.P., A Arias C. (2026). Genomic and clinical features associated with reduced susceptibility to cefiderocol and cross-resistance to β-lactam/β-lactamase inhibitors in a global cohort of *Pseudomonas aeruginosa*. J. Infect. Dis..

[93] Prescott H.C., Antonelli M., Alhazzani W., Møller M.H., Alshamsi F., Azevedo L.C.P., Belley-Cote E., De Waele J., Derde L., Dionne J.C. (2026). Surviving Sepsis Campaign: International guidelines for management of sepsis and septic shock 2026. Crit. Care Med..

[94] Pina-Sánchez M., Rua M., Candel F.J., del Pozo J.L. (2025). Therapeutic optimization against bacteria carrying metalloenzymes: The battle for class B. Rev. Esp. Quimioter..

[95] Vena A., Schenone M., Corcione S., Giannella M., Pascale R., Giacobbe D.R., Muccio M., Mornese Pinna S., Pancaldi L., Mikulska M. (2024). Impact of adequate empirical combination therapy on mortality in septic shock due to *Pseudomonas aeruginosa* bloodstream infections: A multicentre retrospective cohort study. J. Antimicrob. Chemother..

[96] Giovagnorio F., De Vito A., Madeddu G., Parisi S.G., Geremia N. (2023). Resistance in Pseudomonas aeruginosa: A Narrative Review of Antibiogram Interpretation and Emerging Treatments. Antibiotics.

